# Global Microarray Analysis of Carbohydrate Use in Alkaliphilic Hemicellulolytic Bacterium *Bacillus* sp. N16-5

**DOI:** 10.1371/journal.pone.0054090

**Published:** 2013-01-10

**Authors:** Yajian Song, Yanfen Xue, Yanhe Ma

**Affiliations:** 1 State Key Laboratory of Microbial Resources and National Engineering Laboratory for Industrial Enzymes, Institute of Microbiology, Chinese Academy of Sciences, Beijing, China; 2 Graduate School of the Chinese Academy of Sciences, Beijing, China; University Medical Center Utrecht, The Netherlands

## Abstract

The alkaliphilic hemicellulolytic bacterium *Bacillus* sp. N16-5 has a broad substrate spectrum and exhibits the capacity to utilize complex carbohydrates such as galactomannan, xylan, and pectin. In the monosaccharide mixture, sequential utilization by *Bacillus* sp. N16-5 was observed. Glucose appeared to be its preferential monosaccharide, followed by fructose, mannose, arabinose, xylose, and galactose. Global transcription profiles of the strain were determined separately for growth on six monosaccharides (glucose, fructose, mannose, galactose, arabinose, and xylose) and four polysaccharides (galactomannan, xylan, pectin, and sodium carboxymethylcellulose) using one-color microarrays. Numerous genes potentially related to polysaccharide degradation, sugar transport, and monosaccharide metabolism were found to respond to a specific substrate. Putative gene clusters for different carbohydrates were identified according to transcriptional patterns and genome annotation. Identification and analysis of these gene clusters contributed to pathway reconstruction for carbohydrate utilization in *Bacillus* sp. N16-5. Several genes encoding putative sugar transporters were highly expressed during growth on specific sugars, suggesting their functional roles. Two phosphoenolpyruvate-dependent phosphotransferase systems were identified as candidate transporters for mannose and fructose, and a major facilitator superfamily transporter was identified as a candidate transporter for arabinose and xylose. Five carbohydrate uptake transporter 1 family ATP-binding cassette transporters were predicted to participate in the uptake of hemicellulose and pectin degradation products. Collectively, microarray data improved the pathway reconstruction involved in carbohydrate utilization of *Bacillus* sp. N16-5 and revealed that the organism precisely regulates gene transcription in response to fluctuations in energy resources.

## Introduction

Due to the diminishing supply of fossil fuel sources, exploiting alternative renewable energy sources has become increasingly necessary. Plant biomass, as a renewable resource, has great potential in bio-energy production [1]–[3]. Cellulose, hemicellulose, and pectin are the three main polysaccharides composing the plant cell wall; their natural degradation is carried out mainly by microorganisms which are either free or exist as part of the digestive system in higher organisms [4], [5]. Because efficient conversion of biomass to fermentable sugars is a key step in bio-energy production, studies on carbohydrates utilization in microorganisms possessing this ability are important [6], [7].

Microorganisms employ different strategies for degrading polysaccharides and utilizing the degradation products. Aerobic fungi such as *Trichoderma* and *Aspergillus* spp. secrete a number of free enzymes, such as cellulases, hemicellulases, and ligninases, which work cooperatively to completely degrade polysaccharides into mono- and disaccharides extracellularly to allow their assimilation [8]–[10]. Some cellulolytic anaerobic fungi and gram-positive bacteria produce efficient multienzyme complexes, referred to as cellulosomes, which contain cellulolytic and hemicellulolytic enzymes and are able to bind crystalline cellulose [11]–[13]. Due to the powerful ability to degrade crystalline cellulose, organization of the cellulosome and expression of relative enzymes on different substrates have been thoroughly examined, particularly in some *Clostridium* spp. [14]–[16]. It has been found that bacteria not containing cellulosome, such as *Bacillus* spp., secrete extracellular enzymes to degrade polysaccharides and transport partial degradation products into the cell for subsequent processing into fermentable sugars [17], [18]. Although numerous extracellular and intracellular hydrolytic enzymes have been identified for this type of microorganism, how these organisms control transcription of enzymes and transport of their degradation products is not well understood [17],[19].

Sugar transport is a crucial step in carbohydrate utilization of bacteria. Typically, for different sugar substrates, bacterial transport systems can be classified into three types: phosphoenolpyruvate-dependent phosphotransferase systems (PTSs), major facilitator superfamily (MFS) transporters, and ATP-binding cassette (ABC) transporters. PTS transporters catalyze phosphorylation of sugar substrates during their translocation across the cell membrane. Each PTS transporter consists of a sugar-specific multidomain enzyme II (EII) and two general energy coupling proteins, enzyme I (EI) and histidine-containing protein (HPr) [20]. MFS transporters are secondary carriers which transport small solutes in response to chemiosmotic ion gradients and are ubiquitous in bacteria, archaea, and eukarya [21]. ABC transporters utilize energy from ATP hydrolysis to carry specific substrates across membranes. ABC transporters that mediate the uptake of sugars and other carbohydrates are classified into two families, carbohydrate uptake transporter 1 (CUT1) and carbohydrate uptake transporter 2 (CUT2). The CUT1 family transporter possesses one extracellular binding protein, two different membrane proteins, and two identical ATP-binding subunits which act as a homodimer. In contrast, the CUT2 family transporter possesses one extracellular binding protein, two identical membrane proteins acting as a homodimer, and one ATP-binding subunit [22]. CUT1 family members have been reported to transport di- or oligosaccharides, but few members of this family involved in the uptake of hemicellulolytic products have been characterized, making it difficult to predict the exact function based on homologous alignment methods [17].

In bacteria, translation of genes coding enzymes involved in the catabolism of less favorable carbon sources are often repressed in the presence of preferential sugars such as glucose or fructose, which is known as carbon catabolite repression (CCR). CCR mediated by catabolite control protein A (CcpA) is widespread in gram-positive bacteria with low GC content. PTS plays an important role in this mechanism. In gram-positive bacteria with low GC content, HPr contains two phosphorylated sites, a histidine residue and a serine residue. The histidine residue is phosphorylated by phospho-EI, while serine residue is phosphorylated by HPr kinase (HPrK). HPrK is activated by fructose-1,6-bisphosphate, which rapidly accumulates in the present of glucose and other favorable sugars. When its serine residue is phosphorylated, HPr binds to the transcriptional regulator CcpA, inducing the binding of the complex to catabolite responsive element (*cre*) sites in the promoter region of target genes, including genes involved in less favorable carbohydrate utilization, preventing their transcription [23], [24], [25].

*Bacillus* sp. N16-5 is an alkaliphile isolated from the sediment of Wudunur Soda Lake in Inner Mongolia, China. It is a gram-positive and facultative anaerobe which can produce various extracellular or intracellular hydrolytic enzymes and exhibits an excellent ability to grow over the pH range of 8.5–11.5 and NaCl concentration of 0–15% [26]. Its extracellular mannanase and pectin lyase with high activity and extreme condition tolerance have been thoroughly examined in our previous studies [27], [28]. Furthermore, this strain has a broad substrate spectrum and exhibits the capability to grow on multiple soluble polysaccharides (e.g., xylan, mannan, and pectin), making it a good model for studying complex carbohydrate utilization of bacteria that do not contain cellulosome. Transcriptional analysis based on microarrays has been shown to be an effective strategy for examining regulatory mechanisms and annotating functional genes [29], [30]. In this study, we conducted global transcriptional analysis of *Bacillus* sp. N16-5 for growth on ten different mono- and polysaccharides that are abundant in plant biomass to examine the sugar uptake systems and catabolic machinery involved in utilization of test substrates as well as to understand soluble complex carbohydrate utilization by bacteria.

## Results

### Carbohydrate utilization patterns

First, the growth of *Bacillus* sp. N16-5 on ten different carbohydrate substrates was tested. Among substrates tested, glucose, fructose, mannose, arabinose, xylose, and galactose are the most abundant monosaccharides in the nature. Galactomannan, xylan, and pectin are polysaccharides widely present in plant biomass. *Bacillus* sp. N16-5 cannot break down insoluble forms of cellulose such as cotton fiber, filter paper, and microcrystalline cellulose, probably due to the crystalline nature of the sugar polymer. However, *Bacillus* sp. N16-5 can use sodium carboxymethylcellulose (CMC) which is a soluble cellulose derivative, so CMC was also chosen as a substrate. Our experimental data showed that the culture grew to a cell density of approximately 1.5×10^9^ cells/mL on glucose, fructose, mannose, arabinose, and xylose, grew to a cell density of nearly 2.0×10^9^ cells/mL on galactomannan, xylan, and pectin, and grew to a cell density of less than 1.0×10^9^ cells/mL on galactose and CMC.

**Figure 1:**
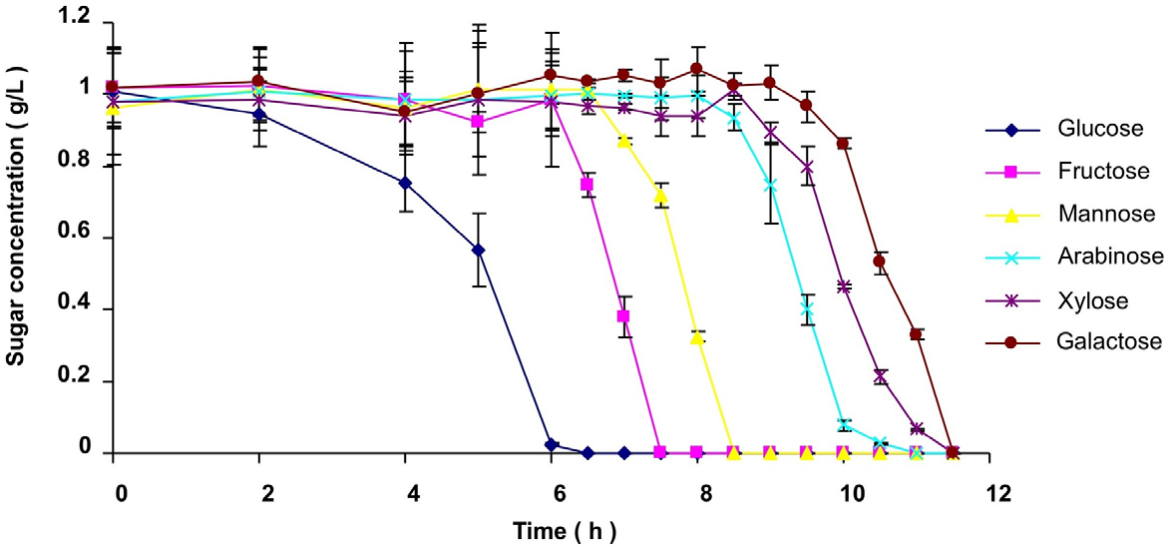
Simultaneous utilization of sugars during growth of *Bacillus* sp. N16-5 in batch cultivation. Equal amounts of each of the six monosaccharides were added into the cultures at the beginning of cultivation. The remaining concentration of each sugar was determined at different time points during the entire cultivation period. Average values at each time point were obtained from triplicate experiments.

The preference of *Bacillus* sp. N16-5 for different pentoses and hexoses was tested by cultivating the cells on a mixture of six monosaccharides. The remaining amount of each monosaccharide was measured, and a clear order of use was observed (Figure 1). Glucose was the first sugar to be consumed, and its presence inhibited utilization of all other sugars. When glucose had been depleted, mannose and fructose were consumed simultaneously, but fructose was consumed faster than mannose. When fructose and mannose had been completely consumed, arabinose, xylose, and galactose were utilized in that order. This hierarchical sugar utilization pattern indicates that carbohydrate metabolism by *Bacillus* sp. N16-5 is under strict control.

### Transcription profiles of *Bacillus* sp. N16-5 grown on different monosaccharides

**Figure 2:**
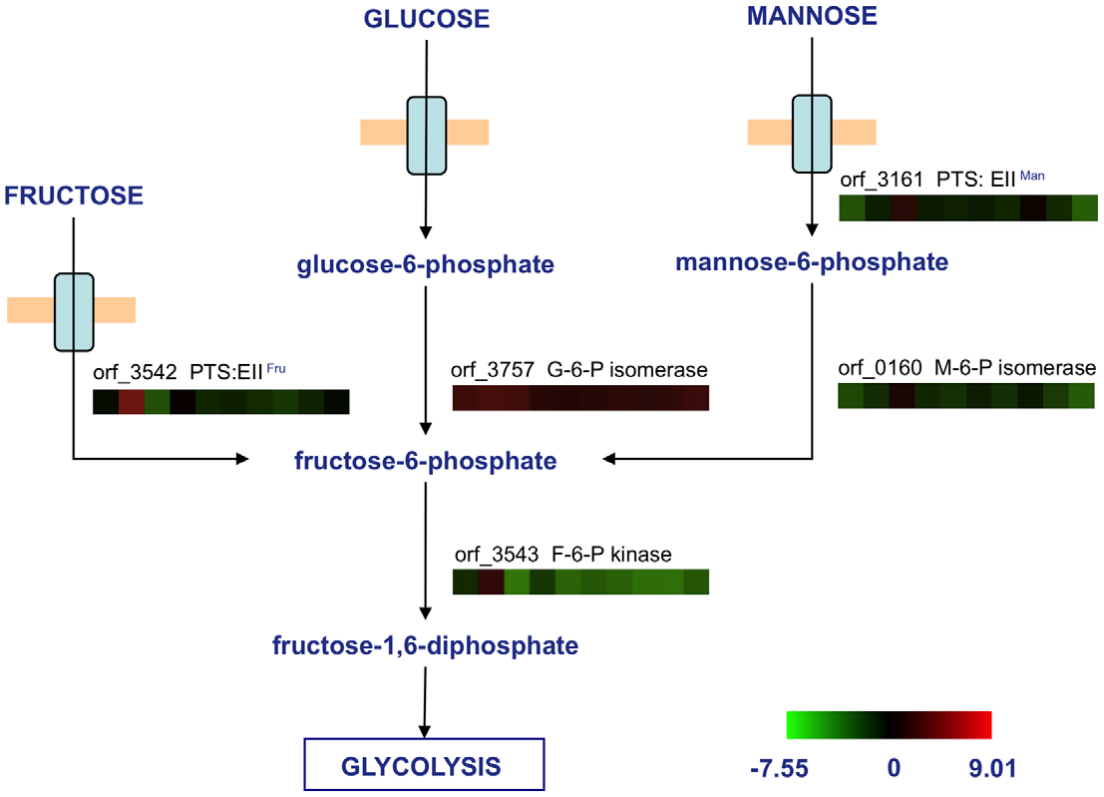
Differential transcription of genes involved in glucose, fructose, and mannose metabolism. Abbreviations: M-6-P, mannose-6-phosphate; F-6-P, fructose-6-phosphate; G-6-P, glucose-6-phosphate. Transcription profiles of specific genes on various substrates were exhibited using color squares in the order of glucose, fructose, mannose, galactose, arabinose, xylose, xylan, galactomannan, pectin, and CMC from left to right. A reference indicating the range of values is located at the bottom of the figure. The value is the log_2_-transformed expression ratio of a specific gene to the mean of all genes in the genome.

**Figure 3:**
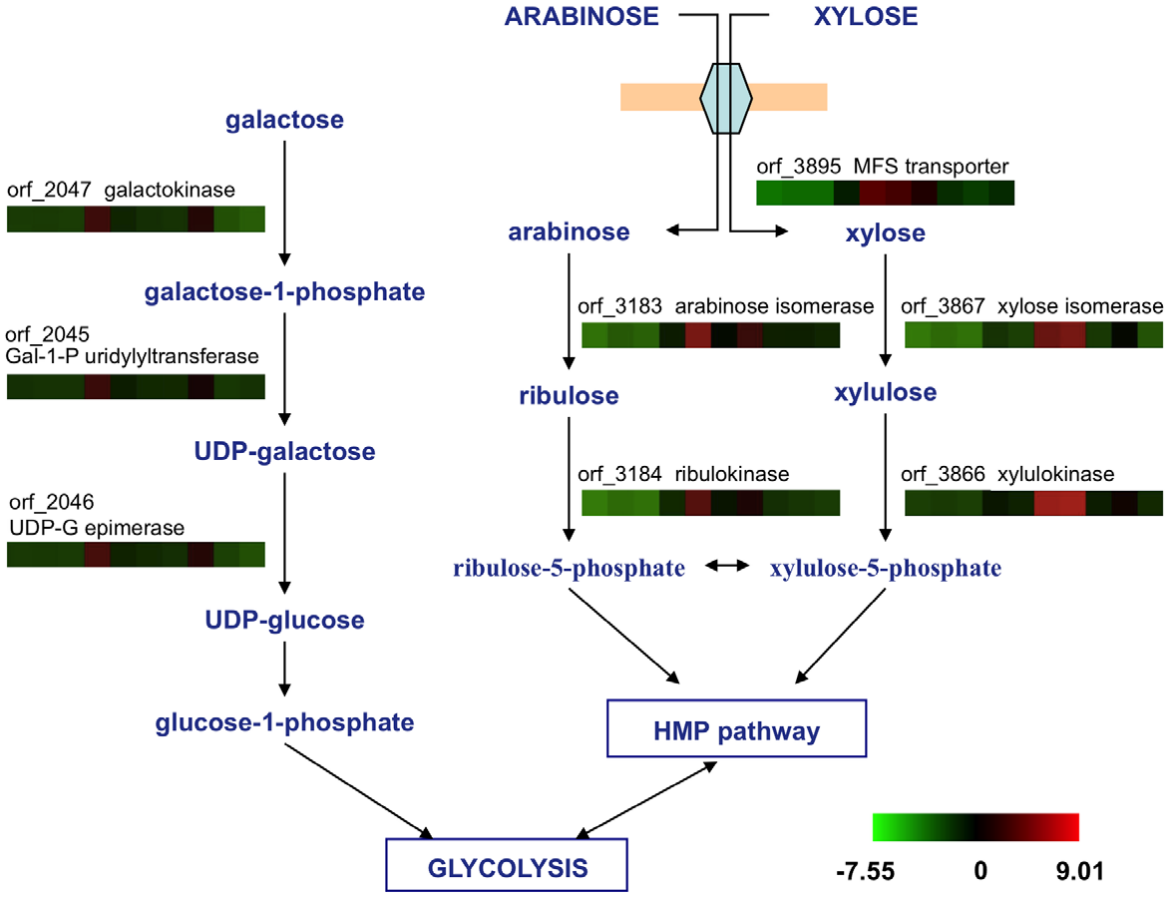
Differential transcription of genes involved in galactose, arabinose, and xylose metabolism. Abbreviations: Gal-1-P, galactose-1-phosphate; UDP-G, uridine diphosphate glucose; HMP: hexose monophophate pathway. Transcription profiles of specific genes on various substrates were exhibited using color squares in the order of glucose, fructose, mannose, galactose, arabinose, xylose, xylan, galactomannan, pectin, and CMC from left to right. A reference indicating the range of values is located at the bottom of the figure. The value is the log_2_-transformed ratio of the transcription level of a specific gene to the mean of all genes in the genome.

One-color microarrays was employed to investigate global gene expression patterns of *Bacillus* sp. N16-5 grown on ten different carbohydrates. All arrays exhibited a percent coefficient of variation (%CV) less than 15 (Dataset S1), indicating the intra-array reproducibility. The results generated for each biological replicate under each condition were compared to the results for samples grown on glucose. The flag of signals, fold changes, and *t*-test results are also shown in Dataset S1. Operons for the transport and metabolism of the six monosaccharides were identified. Gene transcription patterns are shown in Figures 2 and 3.

The fructose operon, which encodes a transcriptional regulator (orf_3541), a PTS system fructose-specific enzyme II (EII^Fru^, orf_3542), and a phosphofructokinase (orf_3543), was identified. The transcription levels of orf_3542 and orf_3543 were approximately 20-fold higher in the presence of fructose than in glucose. Similarly, the mannose operon, which encodes a transcriptional regulator (orf_3159), a PTS system mannose-specific enzyme II (EII^Man^, orf_3161), and a mannose-6-phosphate isomerase (orf_0160), was identified. The transcription levels of orf_3161 and orf_0160 were up-regulated by 39- and 28-fold, respectively, in the presence of mannose compared to glucose.

The operon for galactose metabolism, which encodes a transcriptional repressor (orf_2044), a galactose-1-phosphate uridylyltransferase (orf_2045), an UDP-glucose 4-epimerase (orf_2046), and a galactokinase (orf_2047), was identified. The induction level of the genes varied from 20- to 45-fold in the presence of galactose compared to glucose. Galactose also strongly induced two other operons; their expression patterns are shown in Figure S1. One operon is related to an alternative fructose metabolism pathway and includes a fructose-bisphosphate aldolase (orf_3054), a PTS system fructose-specific enzyme II (orf_3055, 3056), a fructose-1-phosphate kinase (orf_3057), and a transcriptional regulator (orf_3058). In this pathway, fructose was transported and phosphorylated into fructose-1-phosphate rather than fructose-6-phosphate before being converted into fructose-1,6-biphosphate. This result strongly suggests that cross-talk occurs between the galactose and fructose metabolism pathways and that galactose is a strong signal for both galactose and fructose utilization. The other highly expressed operon encodes a glycoside hydrolase (orf_0347), two β-galactosidases (orf_0348, 0350), a LacI family transcriptional regulator (orf_0349), and a CUT1 family ABC transporter (orf_0351–0353).

The operon for arabinose metabolism was identified and found to be up-regulated in the presence of arabinose by 194- to 604-fold compared to glucose. The operon encodes an arabinose utilization protein (orf_3181), an α-L-arabinosidase (orf_3182), an L-arabinose isomerase (orf_3183), a ribulokinase (orf_3184), and a ribulose-5-phosphate 4-epimerase (orf_3185). Arabinose also induced another operon encoding an endo-1,5-α-arabinosidase (orf_2831), a transcriptional repressor (orf_2832) and a CUT1 family ABC transporter (orf_2834–2836) (Figure S1).

Xylose metabolism pathway genes encoding xylulokinase (orf_3866) and xylose isomerase (orf_3867) were found to be highly transcribed in the presence of xylose, with induction levels of 290- to 553-fold compared to glucose. Additionally, an MFS family transporter (orf_3895) showed dramatically increased transcription in the presence of arabinose and xylose compared to other test monosaccharides, suggesting its possible function in pentose uptake. Compared to glucose, the induction levels of this gene on arabinose and xylose are 239- and 168-fold, respectively. Comparing the microarray data of glucose to that of other test substrates, no putative glucose operon was identified.

### Transcription profiles of *Bacillus* sp. N16-5 grown on different polysaccharides

**Figure 4:**
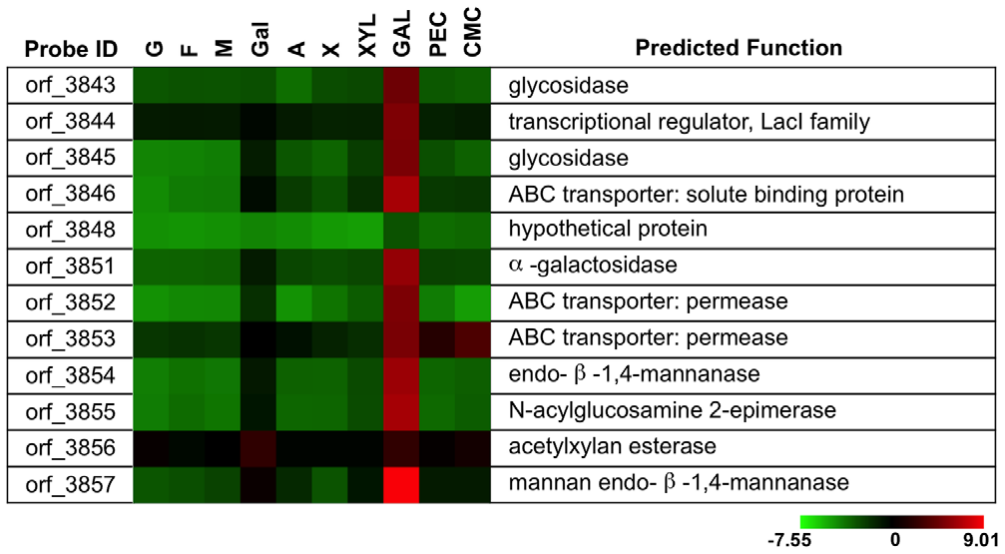
Differential transcription of genes involved in galactomannan utilization. Abbreviations: G, glucose; F, fructose; M, mannose; Gal, galactose; A, arabinose; X, xylose; XYL, xylan; GAL, galactomannan; PEC, pectin. A reference indicating the range of values is located at the bottom of the figure. The value is the log_2_-transformed ratio of the transcription level of a specific gene to the mean of all genes in the genome.

A 17-kb gene cluster containing 12 genes (orf_3843–3857) was observed to be highly transcribed only in the presence of galactomannan (Figure 4). Neither galactose nor mannose, the main components of galactomannan, acted as inducers. Functions of enzymes in this cluster were predicted based on sequence homology analysis. Orf_3851 likely encodes an α-galactosidase from glycoside hydrolase family 31 (GH31), which shares 45% sequence identity with α-galactosidase from *Helianthus annuus* (GI: 29335747). Orf_3854 likely encodes an endo-β-1,4-mannanase of GH103, which shares 45% sequence identity with endo-β-1,4-mannanase (GI: 197305048) from *Alicyclobacillus acidocaldarius*. Orf_3856 likely encodes a polysaccharide esterase from carbohydrate esterase family 7 (GE7), which shares 60% sequence identity with acetyl xylan esterase (GI: 268612269) from *Thermoanaerobacterium* sp. JW/SL YS485. Orf_3843 and orf_3845 were predicted to encode putative glycosidases. Proteins destined towards the secretory pathway contain signal peptide. Prediction using SignalP 3.0 showed that all enzymes in this cluster had no signal peptide sequence except for endo-1,4-β-mannosidase (orf_3857), implying that most enzymes encoded by this cluster act in the cytoplasm. A CUT1 family ABC transporter (orf_3846, 3852, 3853), which is probably involved in uptake of galactomannan degradation products, was also found in this cluster.

**Figure 5:**
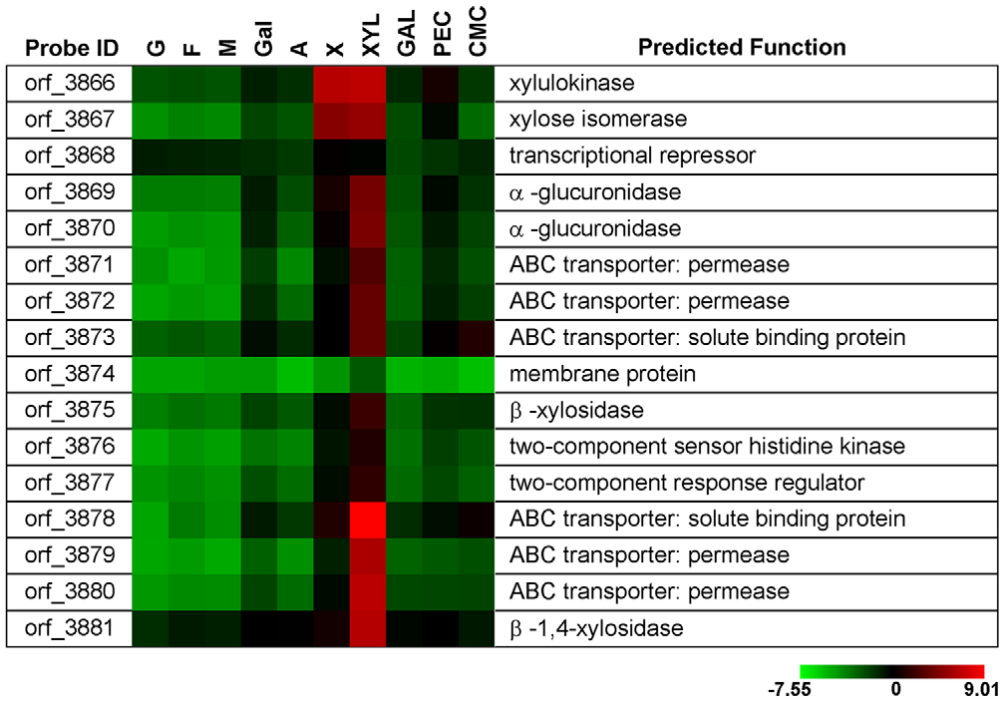
Differential transcription of genes involved in xylan utilization. Abbreviations: G, glucose; F, fructose; M, mannose; Gal, galactose; A, arabinose; X, xylose; XYL, xylan; GAL, galactomannan; PEC, pectin. A reference indicating the range of values is located at the bottom of the figure. The value is the log_2_-transformed ratio of the transcription level of a specific gene to the mean of all genes in the genome.

A strong correlation of gene regulation was observed between cells grown on xylose and xylan. The transcription of a 21-kb gene cluster (orf_3866–3881) was induced by both substrates to a different extent (Figure 5). Transcription of orf_3866 (xylulokinase) and orf_3867 (xylose isomerase) were dramatically up-regulated to a similar extent in the presence of both xylan and xylose. Genes encoding putative xylan degradation enzymes and two CUT1 family ABC transporters (orf_3871–3873, orf_3878–3880) showed significantly higher transcription in the presence of xylan than in the presence of xylose. The function of enzymes in this cluster was predicted based on sequence homology analysis. Orf_3869 and orf_3870 showed the highest identity with two glucuronidases (GI: 37926810, GI: 24987452) from *Geobacillus stearothermophilus*, with sequence identities of 70% and 42%, respectively, while orf_3875 and orf_3881 shared high identity with two β-xylosidases (GI: 82408136, GI: 93279336) from *G. stearothermophilus*, with sequence identities of 60% and 52%, respectively. Signal prediction analysis showed that no enzymes in this operon contain a signal peptide sequence, suggesting that these enzymes are intracellular.

**Figure 6:**
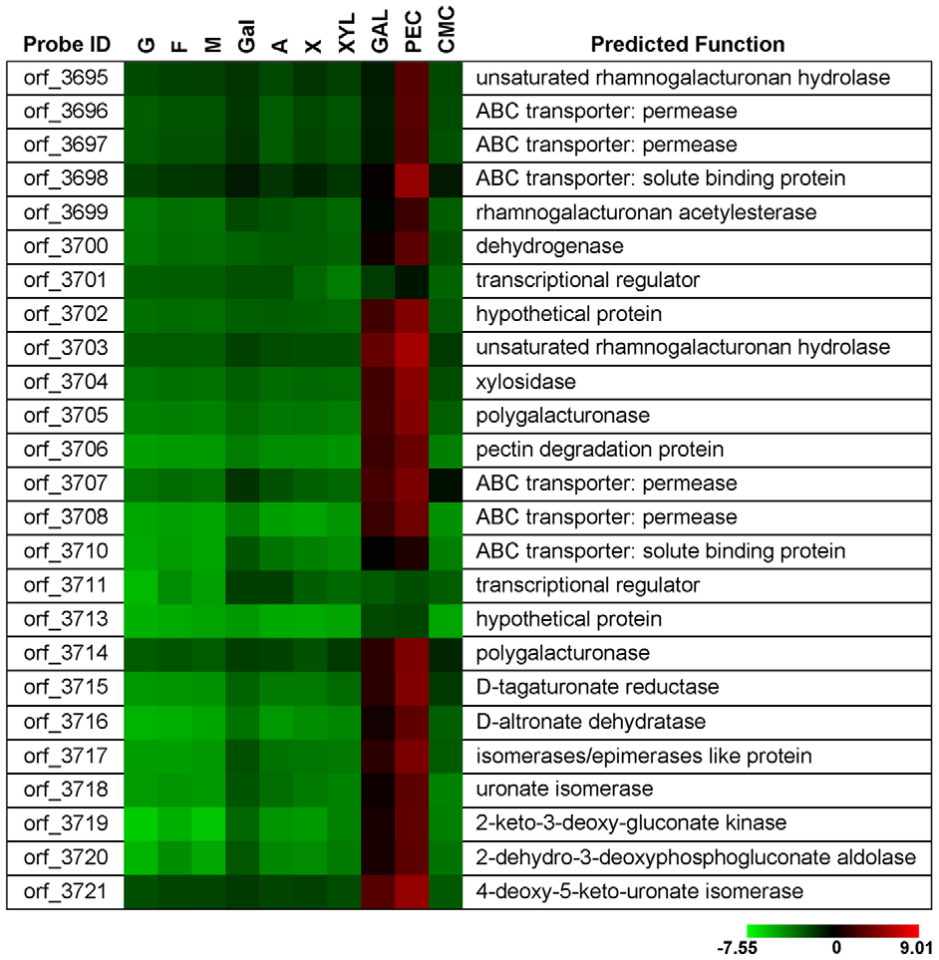
Differential transcription of genes involved in pectin utilization. Abbreviations: G, glucose; F, fructose; M, mannose; Gal, galactose; A, arabinose; X, xylose; XYL, xylan; GAL, galactomannan; PEC, pectin. A reference indicating the range of values is located at the bottom of the figure. The value is the log_2_-transformed ratio of the transcription level of a specific gene to the mean of all genes in the genome.

In the presence of pectin, transcription of a 32-kb gene cluster containing 25 genes (orf_3695–3721) was strongly induced. The transcription patterns and functional annotations of the genes are summarized in Figure 6. Two CUT1 family ABC transporters (orf_3696–3698, orf_3707, 3708, 3710), two regulators (orf_3701, 3711), six pectin degradation enzymes, and seven uronate metabolism enzymes were present in this set of genes. The enzymes encoded by orf_3695 and orf_3703 were predicted to be members of the hydrolase family of GH105. This family contains only unsaturated rhamnogalacturonyl hydrolases, which act specifically on unsaturated rhamnogalacturonan and release unsaturated galacturonic acid. The enzymes encoded by orf_3705 and orf_3714 likely belong to the hydrolase family of GH28. Enzymes in this family are all pectin hydrolases, such as endo- or exo-acting polygalacturonases and rhamnogalacturonan hydrolases. Orf_3704 and orf_3699 were found to encode a putative xylosidase of GH43 and a putative rhamnogalacturonan acetylesterase of GE12, respectively. Orf_3715–3721 are likely to encode a series of enzymes involved in the isomerase pathway of galacturonic acid catabolism [31]. According to signal peptide prediction, all enzymes are intracellular.

*Bacillus* sp. N16-5 exhibits slower growth rate as well as lower final cell density on CMC compared to other polysaccharides tested, which is probably due to the lack of efficient hydrolytic enzymes, uptake systems, or/and mechanism for removing the carboxymethyl groups from glucose. Indeed, unlike galactomannan, xylan, and pectin, no gene cluster or transporter responding specifically to CMC was identified in the transcriptomic analysis. *Bacillus* sp. N16-5 produced a limited number of extracellular enzymes for polysaccharides degradation. We scanned the entire genome of *Bacillus* sp. N16-5 and found only five extracellular enzymes involved in galactomannan, xylan, pectin, and CMC degradation, including a pectate lyase (orf_2241), a family 10 xylanase (orf_4167), two cellulases (orf_1390, 1392), and an endo-β-1,4-mannanase (orf_3857). These enzymes likely act as extracellular endo-acting enzymes for depolymerizing polysaccharides. The Endo-β-1,4-mannanase (orf_3857) is up-regulated in the present of galactomannan as mentioned previously. While, the other four enzymes were consistently and highly transcribed on all substrates except glucose, mannose, and fructose (Figure S1).

### Glucose represses transcription of putative polysaccharides utilization genes

**Figure 7:**
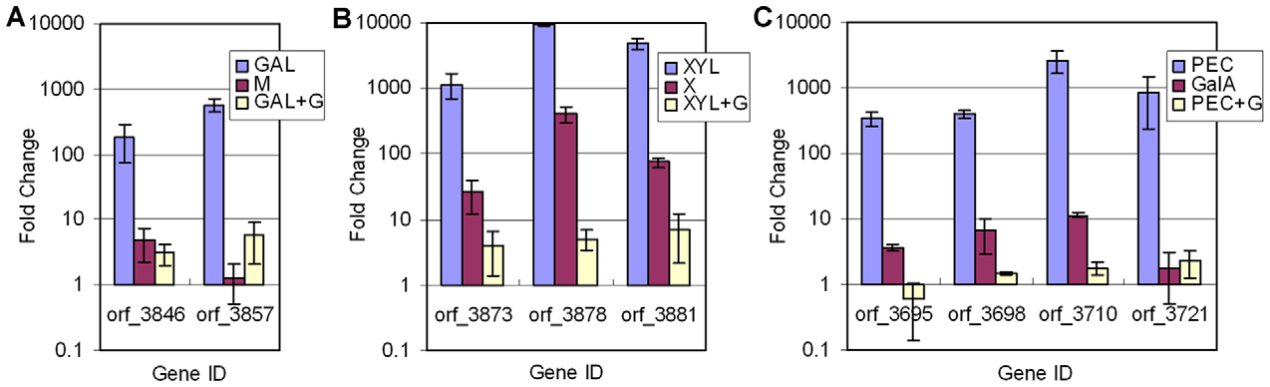
Real-time PCR results for selected genes related to polysaccharide utilization controlled by CCR. Abbreviations: G, glucose; M, mannose; X, xylose; GalA, galacturonic acid; GAL, galactomannan; XYL, xylan; PEC, pectin. Vertical bars represent the fold change of the transcription level of a specific gene in the presence of various carbohydrate sources versus that on glucose.

The relative transcription levels of nine genes on different substrates were determined using real-time PCR (Figure 7). Orf_3846 and 3857 were selected from the putative galactomannan utilization gene cluster. Their transcription was dramatically induced by galactomannan but repressed when the culture was grown on a mixture of glucose and galactomannan. Orf_3873, 3878, and 3881 were selected from the putative xylan utilization gene cluster. Their transcription was dramatically induced by xylan but repressed when the culture was grown on a mixture of glucose and xylan. Similarly, orf_3698, 3710, 3721, and 3695, selected from the putative pectin utilization gene cluster, also showed low transcription level when the mixture of glucose and pectin was taken as the substrate. This result demonstrates that glucose significantly inhibits the transcription of putative gene clusters involved in polysaccharide utilization. Additionally, the transcription levels of the selected genes identified using real-time PCR and microarray experiments had a correlation coefficient of 0.83, indicating a strong positive association between the two sets of data.

## Discussion

The combination of microarray data with gene neighborhood and sequence analysis has been widely used in predicting gene function and analyzing metabolic pathways involved in carbohydrate utilization [29], [30], [32]–[35]. In this study, we carried out global transcriptional analysis of *Bacillus* sp. N16-5 by growing the cells on 10 different mono- and polysaccharides to identify genes related to carbohydrate utilization and to gain insight into its carbon catabolite mechanism. A one-color microarray strategy was employed because this strategy enables easy and flexible comparison of any two sample groups from multiple parallel conditions [36].

Putative operons for fructose, mannose, arabinose, xylose, and galactose, which contain regulators, metabolism enzymes, and transporters, were identified and their metabolic pathways were reconstructed (Figure 2, Figure 3). Candidate transporters were also identified based on global transcriptional analysis. Fructose and mannose were predicted to be transported by PTS transporters, while xylose and arabinose were likely to be transported by an MFS transporter. Transcription of some monosaccharide operons can be triggered by certain polysaccharides containing such monosaccharide residues. For instance, the operon for galactose metabolism was highly transcribed when the culture was grown on galactomannan, and operons for arabinose and xylose were highly transcribed in the presence of xylan. We inferred that specific monosaccharide can be released by polysaccharide degradation, and further induce the transcription of certain operons.

Monosaccharide operons have been widely studied in bacteria, while relatively limited information is available for operons related to polysaccharide utilization. In this study, three gene clusters were found to respond to galactomannan, xylan, and pectin according to polysaccharide transcriptomic analysis, and all of them contain multiple enzymes annotated as polysaccharide degradation-related. Polysaccharides in nature show high flexibility in composition and structures, and their degradation requires the collaboration of multiple enzymes. For example, complete degradation of galactomannan requires of the activity of mannanases, mannosidase, galactosidase, acetyl esterases, and other glycosidase [37]. The transcription of cluster orf_3843–3857 was strongly triggered by galactomannan, and all enzymes encoded by the cluster are annotated as galactomannan degradation-related, strongly suggesting its functional assignment as a galactomannan utilization gene cluster. Similarly, the transcription of cluster orf_3866–3881 was specifically up-regulated in the presence of xylan, and all enzymes encoded by it are annotated as xylan degradation- and xylose metabolism-related, so it is likely to be a xylan utilization gene cluster. The transcription of cluster orf_3695–3721 was triggered by pectin, and all enzymes encoded by it are annotated as pectin degradation- and galacturonic acid metabolism-related, so this cluster is likely to be involved in pectin utilization.

**Figure 8:**
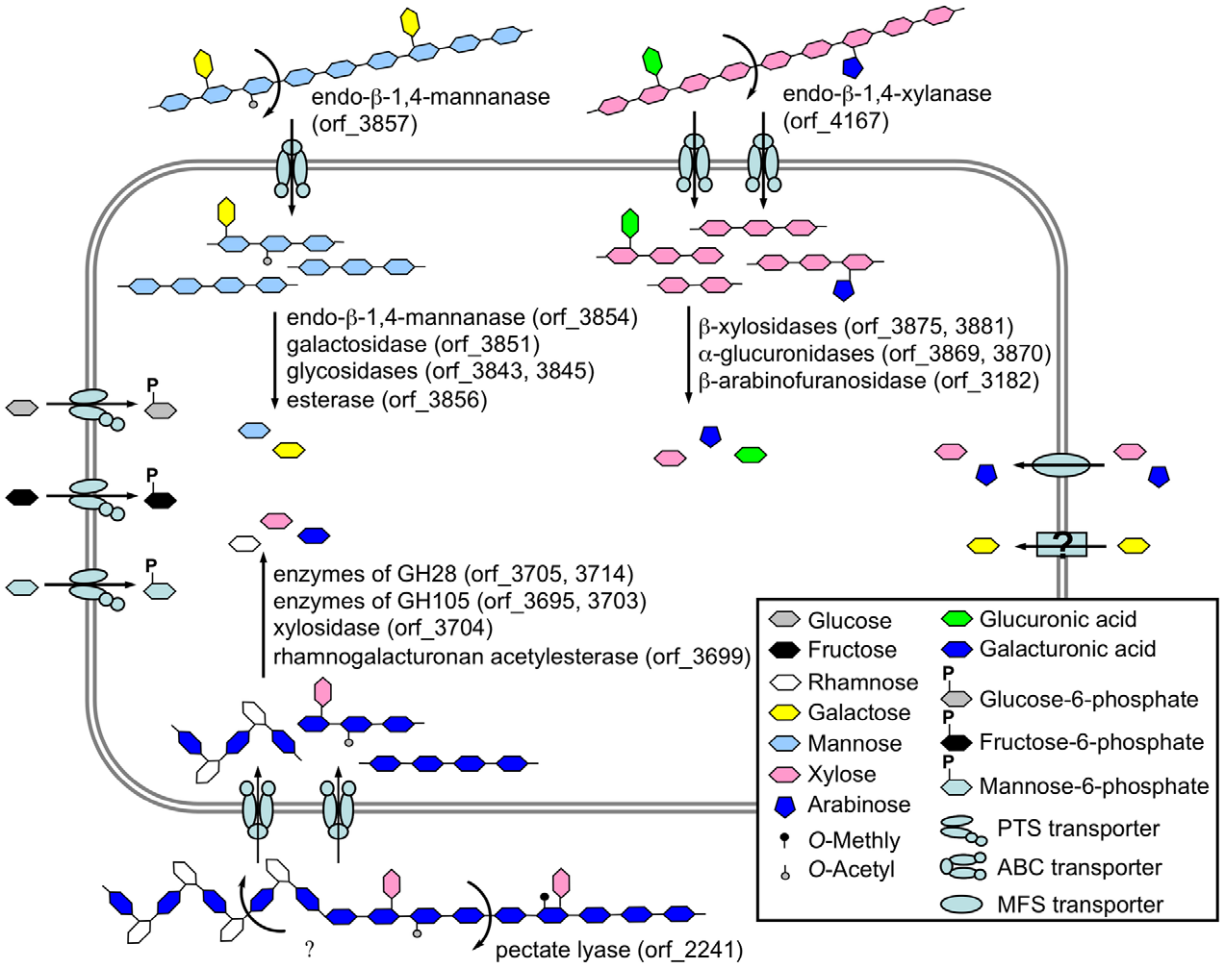
Profile of carbohydrate degradation and transport of *Bacillus* sp. N16-5.

According to function annotation and signal peptide prediction of enzymes in putative polysaccharide gene clusters, the metabolic pathways of galactomannan, xylan, and pectin were reconstructed and the hypothetical carbohydrate utilization profiles of the strain was proposed (Figure 8). In *Bacillus* sp. N16-5, most polysaccharides degradation enzymes are intracellular, suggesting that this organism takes up partially polysaccharide degradation products and then processes them into fermentable sugars in the cell. The five transporters identified in the putative polysaccharide gene clusters likely transport oligomers produced by corresponding polysaccharides degradation since all of them are CUT1 family ABC transporters which are responsible for di- or oligosaccharides uptake. The oligosaccharide transport strategy makes the organism degrade oligomers in the cytoplasm instead of in the environment outside the cell, which may greatly reduce quantity demands on enzymes and speed up the degradation process. Meanwhile, this strategy would also help the organism obtain more carbohydrate resource when in competition with other strains without oligosaccharide transporters. Additionally, no ABC transporter identified in this study contains an ATPase-encoding gene. Because ATPase subunits of the CUT1 family have been confirmed to be functionally exchangeable in heterologous transport systems [38], [39], we presumed that several oligo-transporters may share one ATPase subunit in *Bacillus* sp. N16-5.

The carbohydrate utilization systems of *Bacillus* sp. N16-5 are not only invoked by specific substrates but also under global control. Sequential utilization by *Bacillus* sp. N16-5 was observed in the monosaccharide mixture. Glucose, the preferential sugar, repressed utilization of all other monosaccharides tested. Real-time PCR results also indicated that the presence of glucose repressed transcription of genes within putative polysaccharide utilization gene clusters. Furthermore, key CCR genes were identified in the genome of *Bacillus* sp. N16-5, and their products, CcpA (orf_2342), Hpr (orf_2467), and Hpr kinase (orf_3818), share 71%, 63%, and 69% sequence identity with *Bacillus subtilis*, respectively. These results imply that *Bacillus* sp. N16-5 is likely to regulate its carbohydrate utilization by CCR, which is widespread in gram-positive bacteria with low GC content.

In summary, carbohydrate utilization patterns of alkaliphilic hemicellulolytic bacterium *Bacillus* sp. N16-5 were studied using transcriptomic analysis. Putative gene clusters for monosaccharide and polysaccharide utilization were identified, contributing to carbohydrate utilization pathway reconstruction. A variety of candidate carbohydrate uptake systems for monosaccharides and oligosaccharides were identified, including PTS, MFS, and ABC transporters. CUT1 family ABC transporters likely play an important role in polysaccharide utilization. Differential gene expression and growth experiments implied the presence of a global CCR regulatory network. Collectively, the results revealed that *Bacillus* sp. N16-5 builds efficient systems to utilize complex carbohydrates and precisely regulates its gene transcription to adapt to carbohydrate source fluctuations in the environment.

## Methods

### Bacterial Strains and media

*Bacillus* sp. N16-5 (CGMCC No. 0369) was isolated previously from sediment of the Wudunur Soda Lake in Inner Mongolia, China. Cells were cultivated in modified alkaline Horikoshi-II medium, which contains 5 g peptone, 1 g K_2_HPO_4_·3H_2_O, 0.2 g Mg_2_SO_4_·7H_2_O, 0.1 g yeast extract, and 5 g carbohydrate substrate per liter. Na_2_CO_3_ solution (10%) was added to the medium after sterilization to maintain the pH of the media at approximately 10. The 10 carbohydrate substrates used included D-glucose, D-fructose, D-mannose, L-arabinose, D-xylose, D-galactose, CMC, xylan (from oat spelt), galactomannan (from locust bean), and pectin (from citrus peel). All carbohydrates were purchased from Sigma (St. Louis, MO). Cultures were grown under aerobic conditions at 37°C while shaking. In the test of mixed sugar utilization, the medium contained 1 g of each monosaccharide per liter.

### Analysis of residual sugar

The cultures were centrifuged at 10,000× *g* for 5 min, and then the supernatants were filtered using 0.22-µm size filters and subjected to analysis. Monosaccharide concentrations were determined using a Dionex HPAEC-PAD (high-performance anion-exchange chromatography with pulsed amperometric detection; Sunnyvale, CA) system equipped with a Carbo-Pac PA1 column (Dionex). The operating temperature was 30°C and the mobile phase was 25 mM NaOH.

### Isolation of RNA

*Bacillus* sp. N16-5 cultures in the middle-log phase on various substrates were collected for extraction of total RNA. Three biological replicates were collected for each condition. Cells were rapidly harvested by centrifugation at 10,000× *g* for 1 min and frozen in liquid nitrogen. Next, sediments were grinded in liquid nitrogen to prevent RNA degradation. Total RNA was isolated using TRIzol (Invitrogen, Carlsbad, CA) from homogenized cells according to the manufacturer's instructions.

### Microarray design and experiment

The microarray was designed using Agilent's online design tool, eArray (https://earray.chem.agilent.com/earray/). Each customized microarray (8×15 k) contained spots in triplicate with 4210 specific 60-mer oligonucleotides representing the 4,210 ORFs of *Bacillus* sp. N16-5. Extracted RNAs were further purified using the RNeasy® Mini Kit (QIAGEN, Hilden, Germany). The quality and quantity of samples were determined and checked by Nanodrop ND-1000 and Agilent 2100 bioanalyzer (Agilent, Santa Clara, CA). The Agilent Quick Amp Kit (Agilent) and amino allyl-dUTP (Ambion, Austin, TX) was used to synthesize cDNA from total RNA samples and subsequently produce the amino allyl modified cRNA. Amino allyl modified cRNAs were purified using the RNeasy® Mini Kit and labeled with Cy3 (Cy3 NHS ester, GE Healthcare, Piscataway, NJ). Labeled cRNAs were then purified using the RNeasy® Mini Kit and fragmented in fragmentation buffer (Agilent). The prepared sample was mixed with the 2× Gene Expression Hybridization Kit (Agilent). Hybridization was performed in an Agilent hybridization chamber (G2534A) for 16 h at 65°C with rotation speed of 10 rpm. After hybridization, the slides were washed in Gene Expression Wash Buffer Kit (Agilent). Finally, arrays were scanned using the Agilent Microarray Scanner System (G2565BA) at a resolution of 5 µm. The array was scanned with 100% and 10% PTM and the two sets of data were combined automatically.

### Microarray data collection and analysis

Feature extraction and image analysis software (Feature Extraction Software, Agilent) was used to locate and delineate and integrate the intensity of each spot in the array. Background correction, data normalization, and quality evaluation was performed using the same software. Spots within microarrays were flagged as P (present), A (absent), or M (marginal) according to the signal quality. The quantile method was used for data normalization [40]. The quality of each array was valued as the %CV of the replicated probes designed for quality control. A low %CV indicated high intra-array reproducibility, and microarrays with %CV less than 15% were eligible for analysis.

Statistical analysis of the microarray data was carried out using the SBC Analysis System (SAS) provided by the Shanghai Biochip Co., Ltd (http://www.ebioservice.com). The core arithmetic of the SAS system is R, which is an open source programming language and software environment for statistical computing and graphics. The normalized data was log_2_-transformed and then averaged to obtain the mean value for each gene under each treatment. To compare different treatments, statistical significance was examined using the *t*-test. Fold change was used to value differential gene expression between two treatments; the formula used to calculate this value was *fold change = 2^|mean1-mean2|^*. Flags of all three replicates of the genes selected to calculate fold change should not be ‘A’ for at least one treatment. Genes that were differently transcribed by at least two-fold and has a *P*-value <0.05 were considered to be up- or down-regulated.

### Bioinformatic analysis of enzymes and sugar transporters

Functional prediction of the transporters and enzymes was carried out by sequence homology searches using blastp in the non-redundant protein sequences (nr) database (http://blast.ncbi.nlm.nih.gov/Blast.cgi). Protein sequences of transporters were also subjected to similarity searches using TransportDB (http://www.membranetransport.org/). Family annotation of the enzymes was performed using sequence alignment with the Pfam database (http://pfam.sanger.ac.uk/) and with the Carbohydrate-Active enZYmes Database using the CAZYmes Analysis Toolkit (http://cricket.ornl.gov/cgi-bin/cat.cgi). SignalP 3.0 (http://www.cbs.dtu.dk/services/SignalP/) was used to analyze the enzyme signal peptides.

### Real-time PCR

Relative quantification of the specific transcript was accomplished by the comparative threshold cycle method using a Mastercycler® ep *realplex* thermal cycler (Eppendorf, Hamburg, Germany) detection system with fluorescein-spiked SYBR green as the fluorophore. The transcription levels of orf_3846 and 3857 were quantified when the culture was grown on media containing 0.5% glucose, 0.5% galactomannan, 0.5% mannose, and a mixture of 0.5% glucose and 0.5% galactomannan. The transcription levels of orf_3873, 3878, and 3881 were quantified when the culture was grown on media containing 0.5% glucose, 0.5% xylan, 0.5% xylose, and a mixture of 0.5% glucose and 0.5% xylan. The transcription levels of orf_3698, 3710, 3721, and 3695 were quantified when the culture was grown on media containing 0.5% glucose, 0.5% pectin, 0.5% galacturonic acid, and a mixture of 0.5% glucose and 0.5% pectin. RNA isolated from cells grown on different substrates was reverse transcribed using PrimeScript® RT reagent Kit (TaKaRa, Shiga, Japan), and the real-time PCR reaction system was prepared using SYBR® Premix Ex Taq^TM^ (TaKaRa). 16S rDNA was chosen as the internal control gene.

### Microarray accession number and gene sequences

Microarray data details are deposited in the Gene Expression Omnibus database (http://www.ncbi.nlm.nih.gov/geo/) under accession number GSE38276. The fold changes in the transcription levels of genes for strains grown on various substrates versus glucose are shown in Dataset S1. All gene and protein sequences involved in the study are shown in Dataset S2.

## Supporting Information

Figure S1 **Differential transcription of other related genes.** Abbreviations: G, glucose; F, fructose; M, mannose; Gal, galactose; A, arabinose; X, xylose; XYL, xylan; GAL, galactomannan; PEC, pectin. A reference indicating the range of values is located at the bottom of the figure. The value is the log_2_-transformed ratio of the transcription level of a specific gene to the mean of all genes in the genome.
(TIF)

Dataset S1 **Results of microarray data analysis.** %CV: the percent coefficient of variation of each microarray; different substrates vs glucose (>2-fold): the fold changes in the transcription levels of genes for the strain grown on specific substrates versus glucose (Genes that were differently transcribed by at least two-fold and have *P*-value <0.05 were considered); different substrates vs glucose (full): the log2 transformed normalization signals and the flags of signal of each biological replicate under each condition, the fold changes in the transcription levels of genes for the strain grown on the specific substrate versus glucose and the *t*-test results.
(RAR)

Dataset S2 **Gene and protein sequences involved in this study.**
(TXT)
