# Supplementary figures and images for: Global Microarray Analysis of Carbohydrate Use in Alkaliphilic Hemicellulolytic Bacterium Bacillus sp. N16-5
Source: PLoS One. 2013 Jan 10;8(1):e54090. doi: 10.1371/journal.pone.0054090 (PMC3542313; doi:10.1371/journal.pone.0054090)

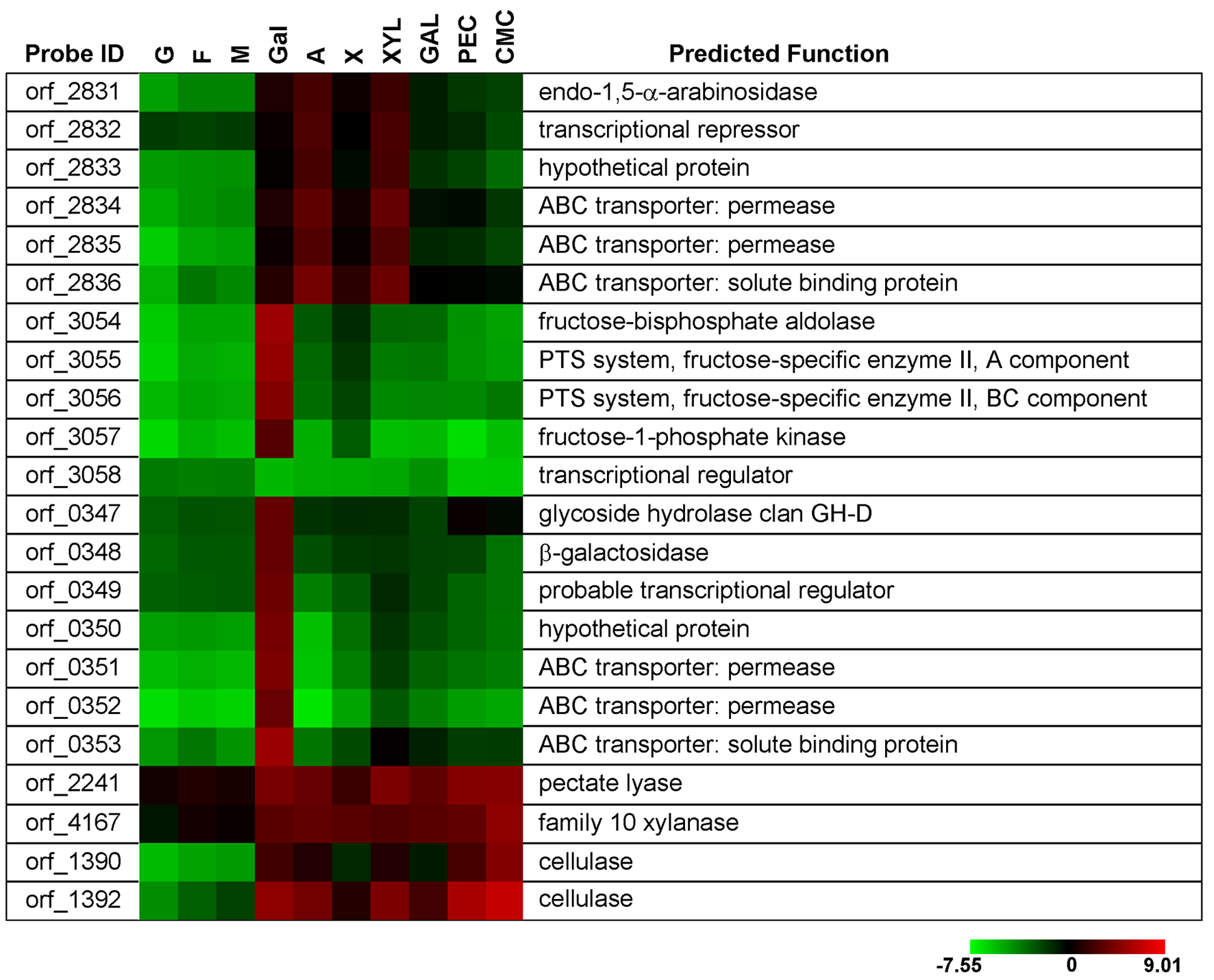

Supplement: Figure S1 — Differential transcription of other related genes. Abbreviations: G, glucose; F, fructose; M, mannose; Gal, galactose; A, arabinose; X, xylose; XYL, xylan; GAL, galactomannan; PEC, pectin. A reference indicating the range of values is located at the bottom of the figure. The value is the log2-transformed ratio of the transcription level of a specific gene to the mean of all genes in the genome. (TIF) [file pone.0054090.s001.tif]
